# Isolation and identification of goose skeletal muscle satellite cells and preliminary study on the function of C1q and tumor necrosis factor-related protein 3 gene

**DOI:** 10.5713/ajas.20.0430

**Published:** 2020-10-13

**Authors:** Han Wang, Ke He, Xuehua Zeng, Xiaolong Zhou, Feifei Yan, Songbai Yang, Ayong Zhao

**Affiliations:** 1 Key Laboratory of Applied Technology on Green-Eco Healthy Animal Husbandry of Zhejiang Province, College of Animal Science and Technology, College of Veterinary Medicine, Zhejiang A&F University, Zhejiang 311300, China

**Keywords:** Goose, Skeletal Muscle Satellite Cells, C1q and Tumor Necrosis Factor-related Protein 3 (CTRP3), Differentiation

## Abstract

**Objective:**

Skeletal muscle satellite cells (SMSCs) are significant for the growth, regeneration, and maintenance of skeletal muscle after birth. However, currently, few studies have been performed on the isolation, culture and inducing differentiation of goose muscle satellite cells. Previous studies have shown that C1q and tumor necrosis factor-related protein 3 (CTRP3) participated in the process of muscle growth and development, but its role in the goose skeletal muscle development is not yet clear. This study aimed to isolate, culture, and identify the goose SMSCs *in vitro*. Additionally, to explore the function of CTRP3 in goose SMSCs.

**Methods:**

Goose SMSCs were isolated using 0.25% trypsin from leg muscle (LM) of 15 to 20 day fertilized goose eggs. Cell differentiation was induced by transferring the cells to differentiation medium with 2% horse serum and 1% penicillin streptomycin. Immunofluorescence staining of Desmin and Pax7 was used to identify goose SMSCs. Quantitative realtime polymerase chain reaction and western blot were applied to explore developmental expression profile of CTRP3 in LM and the regulation of CTRP3 on myosin heavy chains (MyHC), myogenin (MyoG) expression and Notch signaling pathway related genes expression.

**Results:**

The goose SMSCs were successfully isolated and cultured. The expression of *Pax7* and Desmin were observed in the isolated cells. The expression of CTRP3 decreased significantly during leg muscle development. Overexpression of CTRP3 could enhance the expression of two myogenic differentiation marker genes, *MyHC* and *MyoG*. But knockdown of CTRP3 suppressed their expression. Furthermore, CTRP3 could repress the mRNA level of Notch signaling pathway-related genes, notch receptor 1, notch receptor 2 and hairy/enhancer-of-split related with YRPW motif 1, which previously showed a negative regulation in myoblast differentiation.

**Conclusion:**

These findings provide a useful cell model for the future research on goose muscle development and suggest that CTRP3 may play an essential role in skeletal muscle growth of goose.

## INTRODUCTION

Skeletal muscle is an important organ for body movement and energy metabolism, accounting for about 40% of the total animal weight [1]. Meanwhile, it’s also a main part of the meat products consumed by the consumers [2]. To promote meat production, we must first fully understand the process of skeletal muscle formation and growth, as well as its regulatory mechanism. Skeletal muscle satellite cells are muscle-derived stem cells, which are located between the basal lamina and sarcolemma of the muscle fibers, with a potential for cell differentiation and proliferation. They are of great significance for the growth, regeneration and maintenance of skeletal muscle after birth [3]. Skeletal muscle satellite cells were first discovered in the frog, subsequently isolated in multiple species such as humans [4], rats [5], bovine [6], sheep [7], chickens [8], ducks [9]. However, currently, few studies have been performed on the isolation, culture and inducing differentiation of goose muscle satellite cells. Besides, the regulatory factors and mechanisms involved during myogenic differentiation of goose muscle satellite cells still need further exploration.

Currently, studies have found that there are many genes which regulate skeletal muscle growth and development, such as muscle cell transcriptional regulator paired box 3/7 (Pax3/7), myogenic regulator factors family genes and myocyte enhancer factor 2 [10–12]. In addition, non-coding RNAs, such as miRNAs [13], are also involved in various stages during skeletal muscle development. Although the molecular mechanism of skeletal muscle development has been widely studied, there are still many new genes whose functions need to be investigated.

The *CTRP3* gene is a member of the C1q and tumor necrosis factor (TNF)-related protein (CTRPs) family and participates in a series of biological regulation processes. In the liver cells, CTRP3 inhibits gluconeogenesis through the AKT serine/threonine kinase (AKT) signaling pathway, thereby regulating glucose metabolism [14]. In the vascular endothelial cells, exogenous CTRP3 can influence ATP synthesis by activating the mitochondrial ROS (mtROS)/peroxisome proliferator-activated receptor-γ coactivator 1α (PGC-1α) pathway [15]. Moreover, the AKT signaling pathway and the mtROS/PGC-1α signaling pathway were both discovered to play an important role in muscle cells [16,17]. Previous studies have shown that the expression of *CTRP3* was high in mouse embryonic skeletal muscle but was seldomly found in adult skeletal muscle. Meanwhile, *CTRP3* was highly expressed during the differentiation of C2C12 cells [18]. However, the potential function of CTRP3 on skeletal muscle development in goose remains unclear. Therefore, the aim of the present study was to isolate and identify goose skeletal muscle satellite cells, then preliminarily investigate the role of CTRP3 on the differentiation of goose skeletal muscle cells.

In this study, we successfully isolated, cultured and identi fied the goose skeletal muscle cell in vitro. Then, we investigated the expression of *CTRP3* in leg muscle (LM) of goose from the embryonic to neonatal stages. And we also proved that *CTRP3* could regulate the expression of several skeletal muscle cell differentiation related genes in goose.

## MATERIALS AND METHODS

### Goose muscle tissues collection

The fertilized goose eggs used in the experiment were provided by Ningbo Langde Agriculture and Animal Husbandry Co., Ltd. (Ningbo, China). The eggs were put in the same incubator and incubated under the same conditions. The first day after hatching was set as E1. Five eggs were randomly removed at each of seven developmental stages, including embryonic stage of 7 days (E7), embryonic stage of 11 days (E11), embryonic stage of 15 days (E15), embryonic stage of 19 days (E19), embryonic stage of 23 days (E23), and embryonic stage of 27 days (E27) and 3 days post-hatching. Samples of LM were collected, snap frozen into liquid nitrogen and transfer to −80°C refrigerator for storage. All animal procedures used in this study were approved by the Ethics Committee for Animal Experiments of Zhejiang A&F University and were performed in accordance with the Guidelines for Animal Experimentation of Zhejiang A&F University (Hangzhou, China).

### Isolation and culture procedures of muscle satellite cells

The 15 to 20 day fertilized goose eggs were disinfected with ethanol to isolate skeletal muscle satellite cells. The LM of the goose embryo was isolated and placed in a petri dish, washed with phosphate buffer solution (PBS) (HyClone, Logan, UT, USA) containing Penicillin-Streptomycin Liquid (Solarbio, Beijing, China) for 3 times and the skin, blood vessels, adipose tissue and connective tissue were removed. Then, the muscle tissues were cut into meat paste, and digested with 0.25% trypsin-ethylenediaminetetraacetic acid (Thermo Fisher, Shanghai, China) at 37°C for 20 min. Dulbecco’s modified eagle medium (DMEM)/F12 (HyClone, USA) containing 15% fetal bovine serum (FBS, Thermo Fisher, China) was added to terminate the digestion. The suspension was filtered through a 70 μm mesh sieve and centrifuged at 1,000 r/min for 8 min at room temperature. The supernatant was discarded, cells were resuspended with DMEM/F12 containing 15% FBS and cultured in the 5% CO_2_ incubator at 37°C. One hour later, the fibroblasts had adhered to the bottom of cell culture flask, while the skeletal muscle satellite cells remained in the supernatant. The cell suspension was inhaled into a new cell petri dish and this process was repeated twice to enrich muscle satellite cells and eliminate fibroblasts. To induce cell differentiation, culture medium was switched to differentiation medium (DM) with 2% horse serum and 1% penicillin streptomycin (HyClone, USA).

### RNA isolation and cDNA synthesis

Total RNA from tissues or cells was obtained using RNAiso reagent and treated with DNase I (Takara, Kyoto, Japan). The concentration and integrity of RNA were measured by spectrophotometer and denatured gel electrophoresis. The PrimeScript first Strand cDNA synthesis kit (Abcam, Shanghai, China) was used to synthesize the cDNAs. The cDNAs were stored at −20°C until use.

### Quantitative real-time polymerase chain reaction

The cDNA was used as the template for quantitative real-time polymerase chain reaction (qRT-PCR) using the EvaGreen 2X qPCR Master Mix kit (abm, Suzhou, China). Each sample was tested in triplicate, and glyceraldehyde-3-phosphate dehydrogenase was used as a reference gene. Differential gene expression analysis was calculated using the 2^−ΔΔCT^ statistical analysis method. The primers were designed using PrimerPremier.5 software (Table 1) and synthesized by Hangzhou Youkang Biotechnology Co., Ltd. (Zhejiang, China).

### Immunofluorescence staining

Immunofluorescence was used to identify the isolated skeletal muscle satellite cells. Briefly, cells grown in 12-well plate were firstly washed with PBS for three times. and fixed with 4% paraformaldehyde for 15 min. After being washed three times with PBS, the cells were permeabilized with 0.25% Triton X-100 per well for 10 min and blocked at 4°C overnight. Afterwards, cells were incubated with 1:100 diluted primary anti-Desmin (Abcam, China) or anti-Pax7 (Abcam, China) for 1 hour at room temperature. After washing, 1:2,000 diluted fluorescent secondary antibody (Thermo Fisher, China) was used to incubate the cells for 1 hour at room temperature. The cells were then washed, added with 4′,6-diamidino-2- phenylindole (Invitrogen, Carlsbad, CA, USA) and incubated for 15 min at room temperature to stain the cell nuclei. In the end, samples were captured using a fluorescence microscope (OLYMPUS, Tokyo, Japan).

### RNA oligonucleotides and transfection

The RNA oligonucleotides, including the CTRP3 siRNAs and the negative control (NC) were all purchased from RiboBio Co., Ltd. (Guangzhou, China). Lipofectamine 3000 (Invitrogen, USA) was used for transfection following the manufacturer s instructions. All oligonucleotides sequences are listed in Table 2.

### Plasmid construct

PcDNA-3.1 *CTRP3* was the expression vector. The primers were designed to amplify the entire coding sequence region of *CTRP3* and were digested with BamHI and EcoRI restriction sites. The primers’ sequences were as follows: 5′-CGCG GATCCATGGCAGAGAAGGATTTCATC-3′and 5′-CCG GAATTCTTACTTGGTTTCAAAGAGA-3′. The PCR product was then cloned into the pcDNA-3.1 vector.

### Western blot

Total proteins of the cells were prepared and treated as previously described [19]. Cells were homogenized in radio immunoprecipitation assay buffer with 1% phenylmethanesulfonyl fluoride and incubated on ice for 30 min to extract total proteins. Proteins were separated on 12% sodium dodecyl sulfate-polyacrylamide gel electrophoresis gels and then transferred onto the polyvinylidene fluoride membranes (Millipore, Bllerica, MA, USA). After blocking with 5% bovine serum albumin, the membranes were incubated with primary antibodies for MyHC (sc-32732; Santa Cruz Biotechnology, Santa Cruz, CA, USA), CTRP3 (ab36870; Abcam, China), β-actin (ab40854; Abcam, USA). Thereafter, the membranes were incubated with secondary antibody for horseradish peroxidase-labeled anti-rabbit/mouse immunoglobulin G (A0208/A0216; Beyotime, Shanghai, China). Finally, the blots were detected through the chemiluminesence detection system (Amersham, Piscataway, NJ, USA) using chemiluminescence (ECL) reagent (Thermo Scientific, Waltham, MA, USA). Image J software was used for gray value analysis.

### Statistical analysis

All results are shown as the mean±standard error of the mean. Each treatment was repeated for three times. Unpaired Student’s t-test was performed to test statistical significance using SPSS 20.0 software. Two-tailed t tests were used in the analysis. p<0.05 was considered as significant.

## RESULTS

### Morphological observation of goose skeletal muscle satellite cells

**Figure 1:**
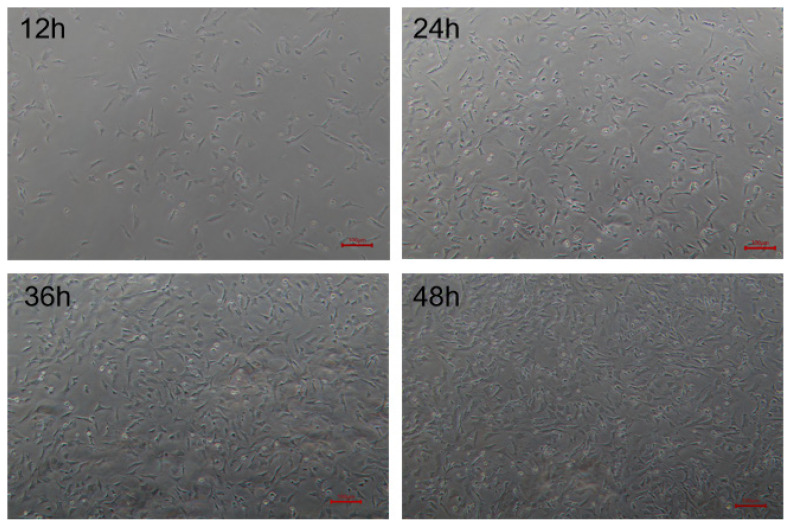
Morphological observation of isolated goose skeletal muscle satellite cells at different time. Cells were observed at 12, 24, 36, and 48 h after culture under microscope with white light (×200).

The isolated goose skeletal muscle satellite cells were cultured in growth medium and began to adhere to plates 12 h later. After 24 h, the cells began to grow dispersedly, and some of them had completely adhered. After 36 h, all the cells were completely adhered and gradually extended into elongated shape. The cell confluence approximately reached 90% after 48 h (Figure 1).

### Identification of the goose skeletal muscle cells

**Figure 2:**
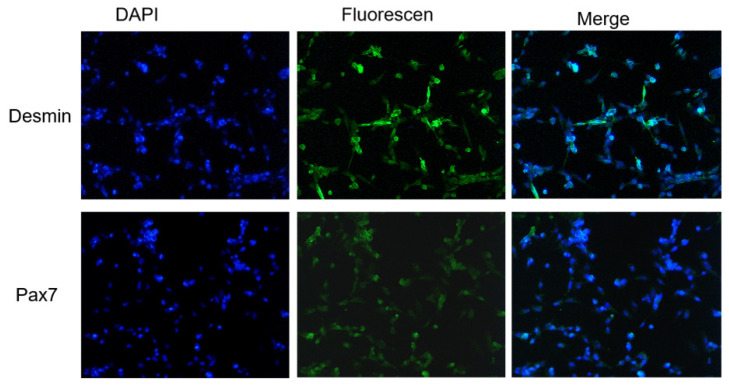
Identification of goose skeletal muscle satellite cells. Desmin and Pax7 were detected with Immunofluorescence; 4′,6-diamidino-2-phenylindole (DAPI) was used to stain the nuclei of goose skeletal muscle cells (×200).

Desmin and Pax7 are special markers of skeletal muscle satellite cells [20], and are often used to distinguish skeletal muscle satellite cell from other cells. After immunofluorescence staining, expression of *Pax7* and Desmin were observed in the isolated cells. *Pax7* was distributed in the nucleus and Desmin was in the cytoplasm (Figure 2). This result further confirmed the isolated cells were goose skeletal muscle satellite cells.

### Developmental expression profile of the goose *CTRP3* in leg muscle

**Figure 3:**
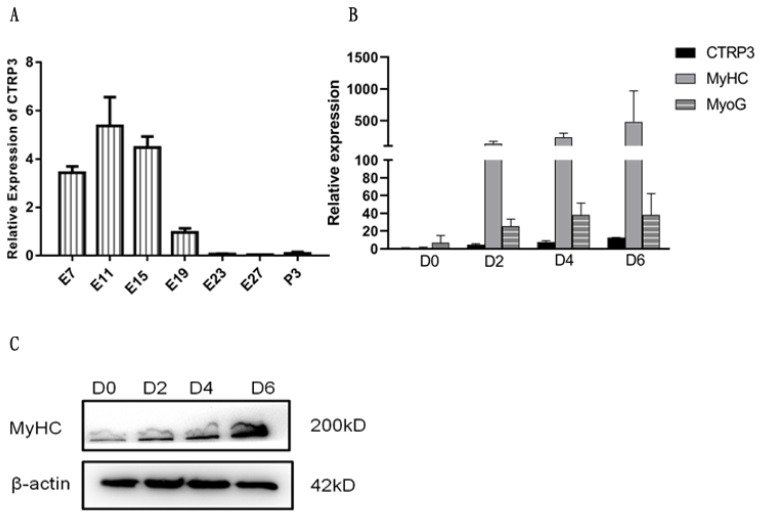
Expression profile of the goose *CTRP3* in LM and the marker genes during myogenic differentiation. (A) The *CTRP3* mRNA expression level was measured by qPCR during leg muscle development of goose. (B) The mRNA level of *MyHC*, *MyoG*, and *CTRP3* in 0, 2, 4, and 6 days of cell differentiation. The fold change was relative to day 0 of DM expression. *GAPDH* was used as the reference gene for Q-PCR. (C) The protein level of MyHC in 0, 2, 4, and 6 days of cell differentiation. β-actin as controls for western blot. Results are expressed as mean±standard error of the mean (n = 3). *CTRP3*, C1q and tumor necrosis factor-related protein 3; LM, leg muscle; qPCR, quantitative polymerase chain reaction; *MyHC*, myosin heavy chains; *MyoG*, myogenin; *GAPDH*, glyceraldehyde-3-phosphate dehydrogenase; DM, differentiation medium; qPCR, quantitative polymerase chain reaction.

To investigate the expression profile of *CTRP3* during LM development, qPCR assay was performed. The mRNA expression level at E19 was regarded as a control and assigned a value of 1. The results show that the expression of *CTRP3* reached its highest level in E11 (Figure 3A). The expression level rose from E7 to E11, then decreased sharply from E15 to P3 (Figure 3A), which indicated that CTRP3 might have a potential role in goose skeletal myogenesis.

Next, we set up a cell model to detect the function of CTRP3 during myoblast differentiation. Both *CTRP3* and two marker genes of myogenic differentiation, *MyHC* and *MyoG*, were up-regulated during this period (Figure 3B–3C). Thus, we could confirm that the *in vitro* cell model was successfully established.

### Knockdown of *CTRP3* attenuates the expression of goose skeletal muscle satellite cell differentiation related genes

**Figure 4:**
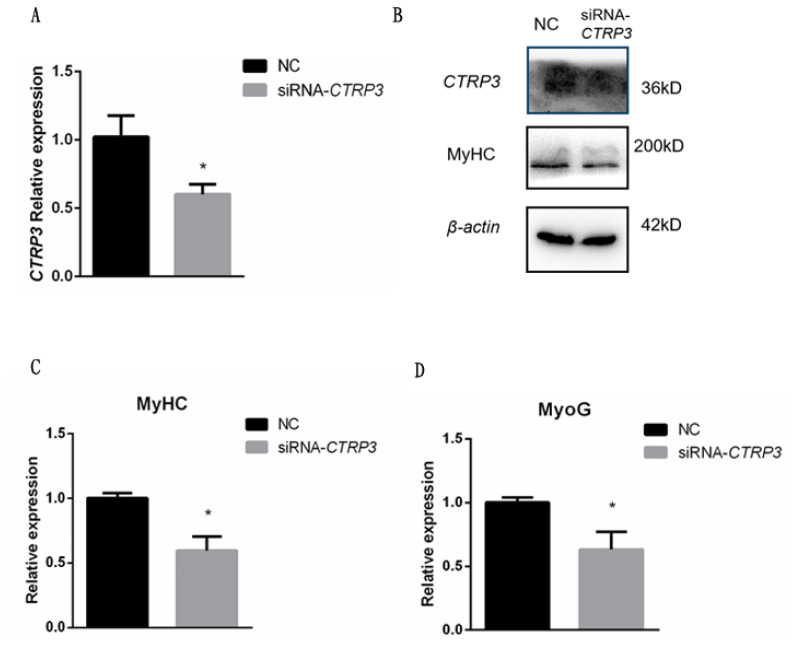
Knockdown of *CTRP3* inhibited the expression of *MyHC* and *MyoG* during differentiation. (A) The mRNA level of *CTRP3* was detected by qPCR in myoblast transfected with siRNA-*CTRP3* or NC at day 3 of DM. (B) The protein levels of CTRP3 and MyHC were detected by western blot in myoblast transfected with siRNA-*CTRP3* or NC at day 3 of DM. (C) The mRNA level of *MyHC* was detected by qPCR in myoblast transfected with siRNA-CTRP3 or NC at day 3 of DM. (D) The mRNA level of *MyoG* was detected by qPCR in myoblast transfected with siRNA-*CTRP3* or NC at day 3 of DM. Results are expressed as mean±standard error of the mean (n = 3). * p<0.05. *CTRP3*, C1q and tumor necrosis factor-related protein 3; *MyHC*, myosin heavy chains; *MyoG*, myogenin; qPCR, quantitative polymerase chain reaction; NC, negative control; DM, differentiation medium.

To assess the role of *CTRP3* gene in goose skeletal muscle satellite cell differentiation, we detected muscle-specific gene expression after transfection with siRNA-*CTRP3*. *CTRP3* was significantly down-regulated in muscle cells at 3 day of DM (Figure 4A–4B). The mRNA level of *MyHC* and *MyoG* were decreased remarkably (Figure 4C–4D), and the protein level of *MyHC* was decreased as well (Figure 4B). Thus, these results indicated that *CTRP3* knockdown might attenuate goose myogenesis.

### Up-regulation of *CTRP3* enhances goose skeletal muscle satellite cell differentiation related genes expression level

**Figure 5:**
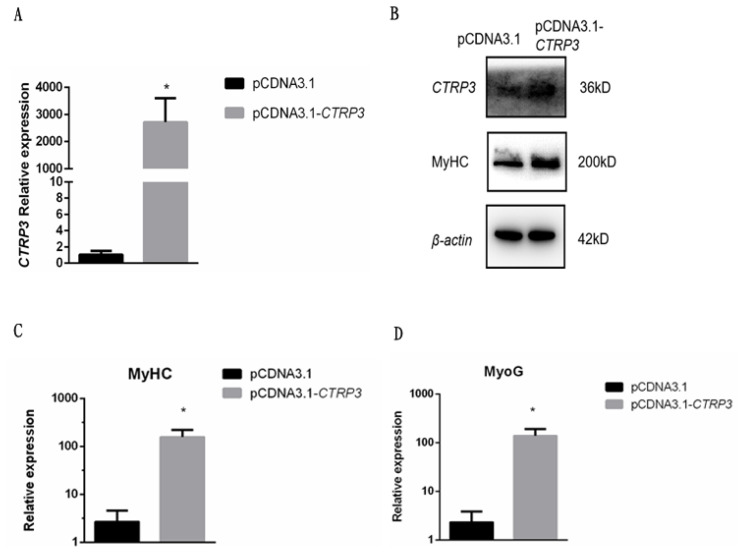
Overexpression of *CTRP3* promoted the expression of *MyHC* and MyoG during differentiation. (A) The mRNA level of *CTRP3* was detected by qPCR in myoblast transfected with pCDNA3.1-*CTRP3* or pCDNA3.1 at day 3 of DM. (B) The protein levels of CTRP3 and MyHC were detected by western blotting in myoblast transfected with pCDNA3.1-CTRP3 or pCDNA3.1 at day 3 of DM. (C) The mRNA level of *MyHC* was detected by qPCR in myoblast transfected with pCDNA3.1-CTRP3 or pCDNA3.1 at day 3 of DM. (D) The mRNA level of MyoG was detected by qPCR in myoblast transfected with pCDNA3.1-CTRP3 or pCDNA3.1 at day 3 of DM. Results are expressed as mean±standard error of the mean (n = 3). * p<0.05. *CTRP3*, C1q and tumor necrosis factor-related protein 3; MyHC, myosin heavy chains; MyoG, myogenin; qPCR, quantitative polymerase chain reaction; NC, negative control; DM, differentiation medium.

To further investigate the role of CTRP3 in goose myoblast differentiation, the cells were transfected with the expression vector pcDNA3.1-*CTRP3*. As a result, the expression of *CTRP3* was much higher than the control at 3 days post-transfection (Figure 5A–5B). The mRNA level of *MyHC* and *MyoG* were increased significantly (Figure 5C-5D). Meanwhile, the protein level of MyHC was also increased (Figure 5B). These results suggested that overexpression of *CTRP3* might induce goose skeletal muscle satellite cell differentiation.

### Effect of *CTRP3* gene on the expression of Notch signaling pathway related genes

**Figure 6:**
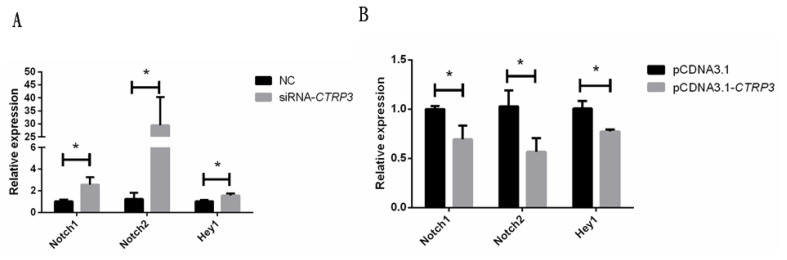
Effect of *CTRP3* gene on the expression of Notch signaling pathway related genes. (A) The mRNA level of *Notch1*, *Notch2*, and *Hey1* was detected by qPCR in myoblasts transfected with siRNA-*CTRP3* or NC at day 3 of DM. (B) The mRNA levels of *Notch1*, *Notch2*, and *Hey1* were detected by qPCR in myoblast transfected with pCDNA3.1-CTRP3 or pCDNA3.1 at day 3 of DM. Results are expressed as mean±standard error of the mean (n = 3). * p<0.05. *CTRP3*, C1q and tumor necrosis factor-related protein 3; *Notch1*, notch receptor 1; *Notch2*, notch receptor 2; *Hey1*, hairy/enhancer-of-split related with YRPW motif 1; qPCR, quantitative polymerase chain reaction; NC, negative control; DM, differentiation medium.

Notch signaling pathway plays an important role in muscle cell differentiation [21]. Previous studies have found that *CTRP3* gene could suppress the expression of Notch signaling pathway during renal cell fibrosis [22]. As shown in Figure 6A, the mRNA level of Notch signaling pathway-related genes, notch receptor 1 (*Notch1*), notch receptor 2 (*Notch2*), and hairy/enhancer-of-split related with YRPW motif 1 (Hey1), were significantly induced after transfecting with siRNA-*CTRP3* in goose skeletal muscle satellite cell. Nevertheless, over-expression of *CTRP3* in DM reduced their expression (Figure 6B). Therefore, we illustrated that CTRP3 might also regulate the Notch signaling pathway in goose skeletal muscle cells.

## DISCUSSION

The content of skeletal muscle satellite cells in the body of animals is related to their age. Studies have shown that the number of skeletal muscle satellite cells continue to decrease as age increase. In adult animals, muscle satellite cells contain only 1% to 5% [23]. Therefore, it is especially important to select the appropriate embryo age to isolate and culture the goose muscle satellite cells. Previous study has found that it was better to isolate skeletal muscle satellite cells from 10 to 18 day embryonic chicks [24]. Therefore, the goose embryos between 15 to 20 days were selected for the isolation of skeletal muscle satellite cells in our study. The results showed that the cells were in a good growth state and had a good differentiation ability, which could meet the later experimental needs.

Pax7 is a specific marker protein of muscle satellite cell and a marker gene in the process of postnatal muscle development. Desmin is a component of the muscle cytoskeleton that prompts the function of skeletal muscle, and also one of the early muscle derived marker proteins [25]. Previous studies showed that only a small proportion of poultry myoblasts expressed desmin in culture. However, desmin was extensively expressed in poultry myotubes. Meanwhile, Pax7 was readily detected in the nuclei of myoblasts but not in myotubes of poultry primary skeletal muscle cultures [26]. In our study, we found that both Desmin and Pax7 were highly expressed in the isolated cells. Therefore, it could be inferred that there might be both myoblasts and differentiated myotubes in our cultured cells, and it fully confirmed that the isolated cells were goose skeletal muscle satellite cells.

According to our previous study, we chose seven stages included embryonic and postnatal period to detect the expression of *CTRP3* [27]. E19 was considered as the fastest growth stage of embryonic muscle of Peking duck [28]. In the present study, we discovered the expression of *CTRP3* decreased sharply from E15 to E19, which indicated that it might have a function in goose skeletal myogenesis. The high expression of *MyHC* and *MyoG* genes were generally regarded as a sign of successful differentiation of skeletal muscle cells [19]. Our results showed that the both genes were up-regulated in the differentiated goose muscle satellite cells, suggesting that our *in vitro* cell differentiation model was successfully constructed. We found that *CTRP3* up-regulated during C2C12 cell differentiation, suggesting that it might play a role in myogenic differentiation.

In our model, the e xpression of *MyHC* and *MyoG* were down-regulated with the knockdown of *CTRP3*. On the other hand, over-expression of *CTRP3* promotes the expression of *MyHC* and *MyoG*. Therefore, CTRP3 might play a positive role in myogenic differentiation. To further investigate the potential mechanism of CTRP3 in goose muscle satellite cell differentiation, we demonstrated that CTRP3 could inhibit the expression of Notch signaling pathway related genes. Activated Notch1 expression in C2C12 myoblasts inhibit muscle cell fusion [29]. Overexpression of Notch2 showed a negative regulation in myoblast differentiation [30]. Besides, Hey1 was found to inhibit myogenesis by repressing expression of key myogenic targets [31]. Thus, we speculated that CTRP3 might regulate the goose skeletal muscle satellite cell differentiation through the Notch signaling pathway. However, further experimental research will be needed to fully explain the relationship between them.

In conclusion, we successfully isolated and cultured goose skeletal muscle satellite cells and established a cell differentiation model *in vitro*. Moreover, our study preliminarily showed that CTRP3 could promote the expression of *MyHC* and *MyoG*, which are two marker genes of goose myogenic differentiation. We also speculated that CTRP3 might regulate the differentiation of goose skeletal muscle satellite cells through the Notch signaling pathway.

## Figures and Tables

**Table 1:** Primers for real-time polymerase chain reaction

Name	mRNA or gene accession number	Sequence (5′→3′)
*MyoG*	NW_013185860.1	F: GAGTTCATTGACTTCGGGATGG R: ATGGAGGAGAGCGAGTGGAG
*MyHC*	KM675469	F: CTCCTCACGCTTTGGTAAAT R: GCTCTGGCTTCTTGTTGGAC
*GAPDH*	NW_013185931.1	F: TCTGTCGTGGACCTGACCTGC R: GCCAGCACCCGCATCAAA
*CTRP3*	NW_013185700.1	F: GTGAATGGGGTGTATTTCTTCACCTT R: GTTTCCCATTCGCAGCCAGACTTC

*MyoG*, myosin heavy chains; *MyHC*, myogenin; *GAPDH*, glyceraldehyde-3-phosphate dehydrogenase; *CTRP3*, C1q and tumor necrosis factor-related protein 3.

**Table 2:** Sequence of RNA oligonucleotides

RNA oligonucleotides	Forward (5′-3′)	Reverse (5′-3′)
Negative control	UUCUCCGAACGUGUCACGU	ACGUGACACGUUCGGAGAA
siRNA-CTRP3	UGGAAACAAUGGAGCAACU	AGUUGCUCCAUUGUUUCCA

CTRP3, C1q and tumor necrosis factor-related protein 3.
